# Effectiveness of Metacognitive Therapy in Patients With Depression and Comorbid Anxiety Symptoms: A Case Series From India

**DOI:** 10.7759/cureus.24229

**Published:** 2022-04-18

**Authors:** Vandita Sharma, Rajesh Sagar, Gaurishanker Kaloiya, Manju Mehta

**Affiliations:** 1 Psychiatry/Clinical Psychology, All India Institute of Medical Sciences, New Delhi, New Delhi, IND; 2 Psychiatry, All India Institute of Medical Sciences, New Delhi, New Delhi, IND

**Keywords:** dysfunctional metacognitive beliefs, worry, rumination, anxiety, depression, metacognitive therapy

## Abstract

Metacognitive therapy (MCT) is a transdiagnostic intervention used to treat different psychiatric disorders. This intervention is based on the concept that persistent emotional distress is a consequence of a particular way of responding to negative thoughts and emotions. MCT for depression and anxiety aims at targeting rumination, worry, and the dysfunctional metacognitive beliefs underlying these thought processes. The purpose of this study was to explore MCT as a treatment for adult patients with depression (either major depressive disorder (MDD) or recurrent depressive disorder, dysthymia, or mixed anxiety depressive disorder) with comorbid anxiety symptoms. Four men diagnosed with depressive disorder with comorbid anxiety symptoms seeking treatment from the outpatient clinic of the Department of Psychiatry at the All India Institute of Medical Sciences, New Delhi, were recruited for the study. Each patient received 10 individual weekly sessions of MCT, lasting up to 1 hour each. Participants were assessed at baseline, post-intervention (right after completing MCT), and at one-month and two-month follow-ups. Primary outcome measures were a reduction in the severity of depression, anxiety, worry, and rumination. Secondary outcome measures were changes in dysfunctional metacognitive beliefs about worry and rumination. All outcomes were assessed via self-report using standardized questionnaires at baseline, post-intervention, one-month, and two-month follow-up. Data for all the outcome measures (at baseline, post-intervention, one month, and two-month follow-up) were plotted on a graph for visual examination. Additionally, we calculated clinically significant change (≥50% reduction in symptom severity and one other standardized criteria for clinically significant changes) for all the primary outcome measures across the above-mentioned four time points. All four men were single, well-educated, and had a mean age of 25.5±4.79 years. Their mean illness duration was 21±0.64 months. None of them had ever received any psychotherapy for their current illness. They had severe depressive and anxiety symptoms at baseline. Three participants had high scores on the rumination and worry scales. At post-intervention, all the participants had significant improvement on all the primary outcome measures, and they maintained their gains on follow-up assessments. Our study generated preliminary evidence supporting the effectiveness of MCT in treating depressive disorders with comorbid anxiety symptoms in the Indian context.

## Introduction

Depressive and anxiety disorders are highly comorbid and are called "first cousins" [1]. A large survey demonstrated that 51% of people with major depressive disorder (MDD) were also diagnosed with an anxiety disorder in the same year, compared to only 11.8% of those without MDD [2]. According to the American Psychiatric Association guidelines [3], the first-line treatment for depression in adults includes either psychotherapy or second-generation antidepressants, and when considering combined treatment, cognitive behavior therapy (CBT) or interpersonal therapy with second-generation antidepressants. Previous research has shown that patients with MDD and comorbid anxiety disorders are more likely to be antidepressant-resistant [4], have high rates of premature termination in psychotherapy [5], and have poor outcomes.

Treatment with first-line antidepressant medications has modest (40-60%) response rates, and low remission rates among patients with MDD and comorbid anxiety [6]. The response rates to CBT among severely depressed patients are only 48-56%, and for less severely depressed patients, 60-65%, based on different assessment criteria [7].

Therefore, there is an urgent need to explore newer, more effective treatments for depressive disorders. Recently, Spijker et al. [8] recommended that transdiagnostic treatments that target the common psychological mechanisms underlying comorbid depression and anxiety may be more efficacious. Metacognitive therapy (MCT) [9] targets the transdiagnostic processes underlying different psychopathologies. It is grounded in the self-regulatory executive function (S-REF) model, which posits that there is a common mechanism underlying most emotional disorders, known as the cognitive attentional syndrome (CAS). The CAS comprises a perseverative thinking style characterized by rumination and worry, increased self-focused attention, threat monitoring, and dysfunctional coping styles like avoidance and thought suppression. Patients with depression and anxiety disorders hold metacognitive beliefs underlying the above processes, which promote the use of these strategies and thereby prolong distress. Studies examining the efficacy of MCT for depressive and anxiety disorders have found it to be highly efficacious, with treatment gains persisting during long-term (up to 3 years) follow-up [10,11]. A study from a tertiary care center in India suggested that mental health professionals were overburdened with patient care [12]. Depressive disorders are highly prevalent among patients visiting these settings [13]. Thus, MCT can be a very valuable therapeutic approach for patients with depressive and anxiety disorders in the above context. The current study aims to explore the effectiveness of MCT in patients with depression and comorbid anxiety symptoms visiting the outpatient psychiatry clinic at a tertiary care center in North India.

Methods

The present study was conducted among adults who were either newly registered or were under follow-up treatment at the outpatient psychiatric clinic at the All India Institute of Medical Sciences, New Delhi, India. We adopted an A-B design with baseline, post-intervention, and follow-up assessments. We contacted those patients who had a provisional diagnosis of a primary mood disorder based on ICD-10 criteria for study participation. Information about the provisional diagnosis was extracted from the medical records. The provisional diagnosis was established by a trained psychiatrist as a part of a routine detailed work-up. The diagnosis of depression was reviewed using the Mini International Neuropsychiatric Interview (MINI) version 6.0 [14]. The International Personality Disorder Examination (IPDE) [15] was administered to assess for Axis II disorders. The eligibility criteria for participation in the study were: 20-45 years of age, at least grade 8 education, primary diagnosis of MDD, moderate or severe without psychotic features, recurrent depressive disorder without psychotic features, or dysthymia or mixed anxiety-depressive disorder based on ICD-10 criteria, moderate to severe depression and anxiety endorsed on self-report measures, Beck’s Depression Inventory-II (BDI-II) [16], and Beck Anxiety Inventory (BAI) [17], respectively, having at least average intellectual capacity, assessed using the Verbal Adult Intelligence scale (VAIS) [18], and being stable on antidepressant medication for at least two months before the study entry. Participants with psychotic symptoms and active suicidal intent or recent past attempts, bipolar disorder, psychotic illnesses, mental and behavioral disorders due to psychoactive substance use, organic psychiatric illnesses, personality disorders, and/or currently receiving or having received a structured psychological intervention for depression in the past year were excluded from the study. Using purposive sampling, four patients (all were men) with depression with comorbid anxiety symptoms were recruited. Written informed consent was obtained from all the participants. The study was approved by the institutional ethics review committee. The participants were assessed at baseline, post-intervention, at one-month, and two-month follow-up.

Outcome measures

Depression severity was assessed using the BDI-II [16], a 21-item self-report measure. The severity of anxiety symptoms was assessed using the 21-item BAI [17]. The Ruminative Response Scale (RRS) [19], which is a 22-item, self-reported measure of ruminative coping responses to depressed mood was used to assess the severity of rumination. The worry severity was assessed using the Anxious Thoughts Inventory (AnTi) [20]. Dysfunctional metacognitive beliefs about worry and rumination were assessed using the Metacognitions Questionnaire (MCQ-30) [21], the Positive Beliefs about Rumination Scale (PBRS) [22], and the Negative Beliefs about Rumination Scale (NBRS).

Treatment program

MCT was delivered following the manualized protocol for the treatment of depression and anxiety as described by Adrian Wells [9]. Ten individually delivered, weekly sessions (45-60 minutes each) were provided to the participants by the first author under the supervision of the last author, who is an experienced senior clinical psychologist with >40 years of clinical experience. In the first two sessions, a case conceptualization using the MCT model was generated and discussed in detail with the participants. Ineffective self-regulatory behaviors were also explored, and the participants were helped with generating an activity schedule to enhance motivation. An attention-training technique was introduced and practiced in the next session. Sessions four and five involved the introduction and practice of detached mindfulness exercises and rumination and worry postponement experiments. The next two sessions focused on challenging the negative and positive metacognitive beliefs about rumination and worry using verbal re-attribution and behavior experiments. Sessions eight and nine focused on identifying and modifying threat-monitoring and unhelpful coping strategies. In the last session, a detailed information processing plan was developed in collaboration with the participants, and fears of symptom recurrence (if any) were addressed. Homework exercises were introduced and reviewed during each session.

## Case presentation

Case A

A 21-year-old unmarried undergraduate working in a software company presented with complaints of sadness, irritability, low appetite, low self-esteem, initial insomnia, and intermittent palpitations that continued for about two years. His symptoms were precipitated by the stress of moving to a big city from his native place. He often compared himself to his colleagues and avoided them. He ruminated about past life events and worried that he would not succeed in the new city. He had changed several jobs due to his symptoms. His rumination and worry had worsened over time and prevented him from falling asleep. He believed that his tendency to think a lot was "not normal" and was contributing significantly to his symptoms. He spent at least four to five hours of his waking time mulling over his problems. He would actively engage in his thoughts about trivial things happening to him, which would trigger anxiety and catastrophic thinking that something was seriously wrong with his body that required investigation. He dwelled a lot on all the possible things he was missing in his life because of worrying. On metacognitive profiling, he endorsed that ruminating helped him stay away from bad company. Excessive thinking would offer a "closure" following which he would be able to focus uninterruptedly on the tasks, considered brooding a sign of "maturity" and equated being thoughtful with being a "good human being." He believed that worrying was an effective coping strategy. He also had strong negative beliefs about the uncontrollability and danger of rumination and experienced negative beliefs about the social and interpersonal consequences of ruminating. He felt that he wouldn’t be able to stop negative thinking, and his tendency to ruminate could turn him into a "failure." He was provisionally diagnosed with mixed anxiety and depressive disorder and was treated with escitalopram, clonazepam, and zolpidem, but had not received any psychotherapy since the onset of his symptoms.

Case B

A 32-year-old unmarried man with a post-graduate education, preparing for a competitive exam, presented with persistent extreme sadness, anhedonia, tiredness, regret over poor exam performance, low self-confidence, hopelessness about his future, and indecisiveness, along with the feelings of low self-worth, self-efficacy, and self-criticism. These symptoms have continued for two and a half years. He had become socially isolated. He was worried that he would not get a job. He would feel an urge to think about his worries. He would spend hours ruminating over his past failures, trying to understand the reasons. On metacognitive profiling, he believed that worrying and ruminating could help him find solutions to his problems and avoid mistakes. He felt his "habit of worrying" helped him become more determined about his life goals. Whenever he worried for a long duration, he experienced palpitations. He believed that ruminating about depressive symptoms would help him find a solution to snap out of depression. He endorsed that thinking more was a sign of being incapable and helpless, and excessive rumination was a sign of a weak personality with excessive thinking "weakening" cognitive capacity. He considered himself a "bad person," fearing that his family and friends would leave him if they came to know about his negative thoughts. He believed that his ruminations were uncontrollable and disabling. He was assigned a provisional diagnosis of mixed anxiety and depressive disorder. He had been on escitalopram and clonazepam for six months without any psychotherapy.

Case C

A 26-year-old unmarried man with a post-graduate education, currently employed, presented with persistent irritability, tiredness, pessimism, excessive worrying, palpitations, low self-worth and self-confidence, poor concentration, initial insomnia, decreased confidence, and indecisiveness. His symptoms started a year ago. He spent excessive time thinking about his weaknesses. Although he felt that worrying was not an effective coping strategy, it helped him perform better. He spent a lot of time brooding about his tiredness, his failures, his miseries, and his shortcomings. He believed that his worries and ruminations were uncontrollable and dangerous for him. He believed that his negative thinking would make him a "failure." He felt guilty for not being able to control his thinkings. He was provisionally diagnosed with MDD and a comorbid anxiety disorder, not otherwise specified. He had been taking escitalopram, mirtazapine, and clonazepam for three months, but had never received psychotherapy.

Case D

A 23-year-old unmarried man with a graduate level education, currently preparing for competitive exams, presented with persistent and severe headaches, low mood, anhedonia, negative thoughts, exhaustion, poor concentration, easy crying, and pessimism. He also had episodes of anxiety with autonomic symptoms. He had been experiencing these symptoms for two years. He spent excessive time on negative thoughts and felt that his mind would never stop working, which would make him tired. He experienced excessive rumination and worry and believed that he would not be able to focus on his present activities unless he concluded his ruminative cycle. Listening to others’ problems triggered his ruminations. He felt that ruminations helped him understand and improve himself, and worrying helped him prevent mistakes. He equated ruminating with being lonely and "bizarre." He felt a strong need to control his thinking. Initially, he took treatment from general physicians and later from a neurologist. About 1.5 years into his illness, he visited our center. He was given a provisional diagnosis of recurrent depressive disorder with a comorbid tension-type headache. He had been on mirtazapine, clonazepam, propranolol, and amitriptyline. He had not received any formal psychotherapy.

## Discussion

Data analysis

The clinical data obtained from the participants at baseline, post-intervention, one month, and two-month follow-up were plotted on a graph for visual examination. A clinically significant change in symptoms across baseline, post-intervention, and follow-up (≥50% reduction in symptom severity) was calculated using the following formula [23]: 𝑃𝑟𝑒𝑠𝑐𝑜𝑟𝑒−𝑃𝑜𝑠𝑡𝑠𝑐𝑜𝑟𝑒/𝑃𝑟𝑒𝑠𝑐𝑜𝑟𝑒 × 100=𝑇ℎ𝑒𝑟𝑎𝑝𝑒𝑢𝑡𝑖𝑐 𝑐ℎ𝑎𝑛𝑔𝑒.

A clinically significant change was also computed using the standard cut-offs for BDI-II and BAI, as reported in other studies. For BDI-II, a cut-off score of ≤14 and a reliable change index of 9 were used [24]. Those participants who achieved a score of ≤14 on the BDI-II at post-intervention and follow-up assessment as well as achieving a reduction of at least 9 points on the BDI-II from baseline were classified as "recovered." Participants not meeting one of these criteria were classified as "improved," and those who did not meet either of these criteria were classified as "unchanged." Similarly, for BAI, a standard cut-off score of ≤10 and a reliable change index of 10 were used [25]. Those participants with a score of ≤10 and who had a reduction of at least 10 points in their raw scores compared to the baseline were classified as "recovered." Those participants who met either one of the two criteria were deemed as "improved," and those who met neither of the criteria were marked as "unchanged." These criteria have also been used in other studies examining the effectiveness of MCT among patients with depressive and anxiety disorders [26,27].

Results and discussion

This study aimed to explore the effectiveness of MCT in reducing symptoms of depression, anxiety, rumination, worry, and dysfunctional metacognitive beliefs among patients experiencing depression with comorbid anxiety symptoms. All the four participants included in this case series were single men, two had post-graduate educations, one had a bachelor’s degree, while one was an undergraduate. Their mean age was 25.5 years (SD=4.79). Two participants had a provisional diagnosis of mixed anxiety-depressive disorder, one had MDD with comorbid anxiety disorder, not otherwise specified, and the fourth participant had a provisional diagnosis of recurrent depressive disorder with comorbid tension-type headache. The mean duration of illness was 21 months (SD = 0.64). At baseline, all the participants had severe depressive and anxiety symptoms. Baseline, post-treatment, and follow-up assessment scores for each participant on BDI-II, BAI, RRS, and AnTi have been presented in the following Tables 1-4, respectively.

**Table 1:** Therapeutic changes in the scores at baseline, post-intervention, one month, and two-month follow-up for depressive symptoms BDI-II: beck depression inventory-II, B/L: baseline, PI: post-intervention, 1 mo f/u: one-month follow-up, 2 mo f/u: two-month follow-up

Patient	BDI-II						
	B/L	PI	1 mo f/u	2 mo f/u	Therapeutic change % B/L to PI	Therapeutic change % B/L to 1 mo f/u	Therapeutic change % B/L to 2 mo f/u
A	32	14	12	12	56.3	62.5	62.5
B	48	20	21	19	58.3	56.3	60.4
C	52	13	10	12	75.0	80.8	76.9
D	42	14	12	13	66.7	71.4	69.0

**Table 2:** Therapeutic changes in the scores at baseline, post-intervention, one month, and two-month follow-up for anxiety symptoms BAI: Beck's anxiety inventory, B/L: baseline, PI: post-intervention, 1 mo f/u: one-month follow-up, 2 mo f/u: two-month follow-up

Patient	BAI						
	B/L	PI	1 mo f/u	2 mo f/u	Therapeutic change % B/L to PI	Therapeutic change % BL to 1 mo f/u	Therapeutic change % BL to 2 mo f/u
A	31	8	7	8	74.2	77.4	74.2
B	47	10	10	9	78.7	78.7	80.9
C	47	9	10	10	80.9	78.7	78.7
D	39	10	9	8	74.4	76.9	79.5

**Table 3:** Therapeutic changes in the scores at baseline, post-intervention, one month, and two-month follow-up for rumination severity RRS: Ruminative Response Scale, B/L: baseline, PI: post-intervention, 1 mo f/u: one-month follow-up, 2 mo f/u: two-month follow-up

Patient	RRS						
	B/L	PI	1 mo f/u	2 mo f/u	Therapeutic change % B/L to PI	Therapeutic change % BL to 1 mo f/u	Therapeutic change % BL to 2 mo f/u
A	33	22	23	25	38.9	36.1	30.6
B	63	24	28	30	61.9	55.6	52.4
C	66	24	26	31	63.6	60.6	53.0
D	65	24	26	29	63.1	60.0	55.4

**Table 4:** Therapeutic changes in the scores at baseline, post-intervention, one month, and two-month follow-up for worry severity AnTi: anxious thoughts inventory, B/L: baseline, PI: post-intervention, 1 mo f/u: one-month follow-up, 2 mo f/u: two-month follow-up

Patient	AnTi						
	B/L	PI	1 mo f/u	2 mo f/u	Therapeutic change % B/L to PI	Therapeutic change % BL to 1 mo f/u	Therapeutic change % BL to 2 mo f/u
A	43	23	25	26	46.5	41.9	39.5
B	58	26	28	31	55.2	51.7	46.6
C	67	26	28	32	61.2	58.2	52.2
D	61	27	31	34	55.7	49.2	44.3

Three participants had high scores at baseline on RRS and AnTi, suggesting that their severity of rumination and worry was high. All the participants had very high scores in the meta-worry domain of AnTi, indicating that they had very strong negative appraisals of the uncontrollability and danger related to worrying. All the participants found it difficult to pull themselves out of their negative thoughts and worries and felt a high need to control their thoughts. Interestingly, according to the metacognitive model [28,29], meta-worry has been closely linked to psychopathology. Elevated scores on meta-worry have also been identified to distinguish patients with depressive and anxiety symptoms from healthy controls [30].

Baseline, post-intervention, and follow-up scores on the standard measures of dysfunctional metacognitive beliefs (MCQ-30, PBRS, and NBRS) have been provided in Tables 5-7, respectively.

**Table 5:** Therapeutic changes in scores at baseline, post-intervention, one month and two-month follow-up for dysfunctional metacognitive beliefs about worry MCQ-30: metacognitions questionnaire-30, B/L: baseline, PI: post-intervention, 1 mo f/u: one-month follow-up, 2 mo f/u: two-month follow-up

Patient	MCQ-30						
	B/L	PI	1 mo f/u	2 mo f/u	Therapeutic change % B/L to PI	Therapeutic change % BL to 1 mo f/u	Therapeutic change % BL to 2 mo f/u
A	47	35	38	41	25.5	19.1	12.8
B	77	33	36	38	57.1	53.2	50.6
C	87	35	39	43	59.8	55.2	50.6
D	70	34	38	43	51.4	45.7	38.6

**Table 6:** Therapeutic changes in scores at baseline, post-intervention, one month and two-month follow-up for positive metacognitive beliefs about rumination PBRS: Positive Beliefs about Rumination Scale, B/L: baseline, PI: post-intervention, 1 mo f/u: one-month follow-up, 2 mo f/u: two-month follow-up

Patient	PBRS						
	B/L	PI	1 mo f/u	2 mo f/u	Therapeutic change % B/L to PI	Therapeutic change % BL to 1 mo f/u	Therapeutic change % BL to 2 mo f/u
A	10	10	11	12	0.0	−10.0	−20.0
B	26	9	10	11	65.4	61.5	57.7
C	29	9	11	12	68.9	62.1	58.6
D	35	11	12	14	68.6	65.7	60.0

**Table 7:** Therapeutic changes in scores at baseline, post-intervention, one month and two-month follow-up for negative metacognitive beliefs about rumination NBRS: Negative Beliefs about Rumination Scale, B/L: baseline, PI: post-intervention, 1 mo f/u: one-month follow-up, 2 mo f/u: two-month follow-up

Patient	NBRS						
	B/L	PI	1 mo f/u	2 mo f/u	Therapeutic change % B/L to PI	Therapeutic change % BL to 1 mo f/u	Therapeutic change % BL to 2 mo f/u
A	24	13	14	17	45.8	41.7	29.2
B	47	15	16	17	68.1	65.9	63.8
C	40	17	18	19	57.5	55.0	52.5
D	39	13	15	17	66.7	61.5	56.4

At the baseline, all the patients also obtained high scores on negative metacognitive beliefs about the uncontrollability and danger of rumination. Three participants had high scores on positive beliefs about rumination. We observed that negative metacognitive beliefs were endorsed more strongly than positive metacognitive beliefs. This finding was consistent with what Rafique [31] had found in an exploratory study of metacognitive beliefs about depressive rumination among women of Pakistani origin. At baseline, three participants also had high scores on the MCQ-30, pointing to their significant dysfunctional metacognitive beliefs about worry. In particular, scores on the negative metacognitive beliefs about danger and uncontrollability of worry and need to control worry subscales of MCQ-30 were very high. All the participants believed that worrying and ruminating were harmful and dangerous because they could make them "go crazy" or turn them into "failures" or lead to some physical sickness. They also had unrealistic expectations about the degree of control they had over their thoughts. They felt it was necessary for them to control all their thoughts all the time, failing which they would become dysfunctional or be punished by God. These negative metacognitive beliefs led to an escalation of worrying or ruminating and emotional distress.

**Figure 1:**
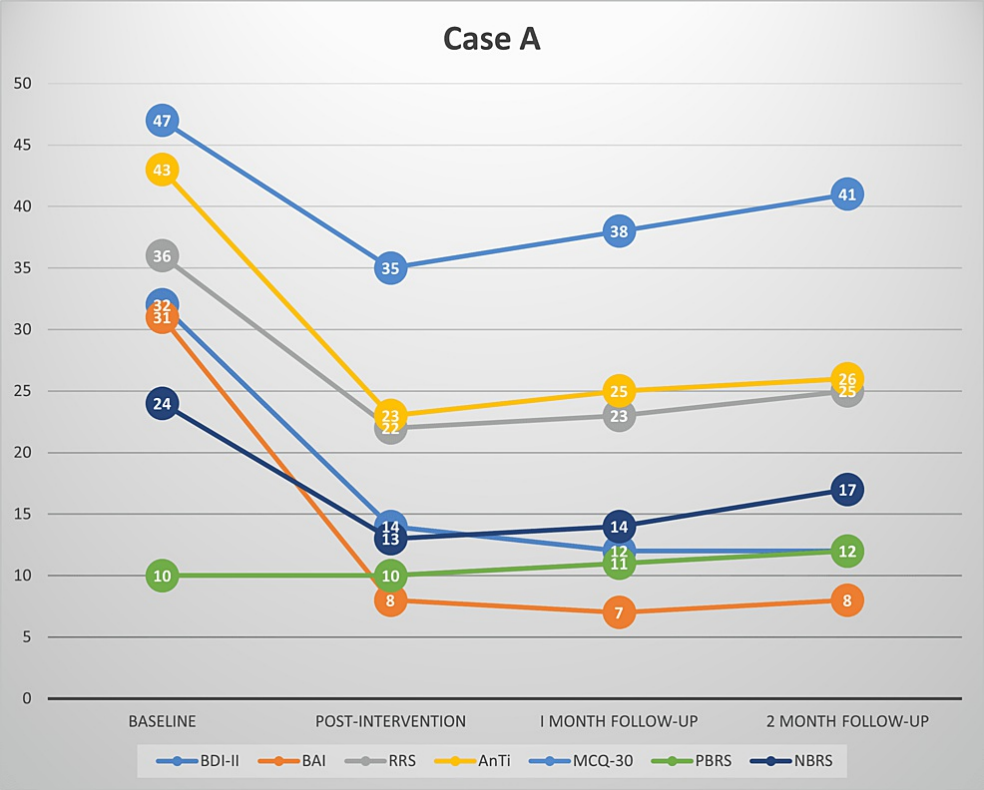
Scores of case A on BDI-II, BAI, RRS, AnTi, MCQ-30, PBRS, and NBRS across baseline, post-intervention, one-month follow-up and two-month follow-up BDI-II: Beck's depression inventory-II, BAI: Beck’s anxiety inventory, RRS: Ruminative Response scale, AnTi: anxious thoughts inventory, MCQ-30: metacognitions questionnaire-30, PBRS: Positive Beliefs about Rumination scale, NBRS: Negative Beliefs about Rumination scale

**Figure 2:**
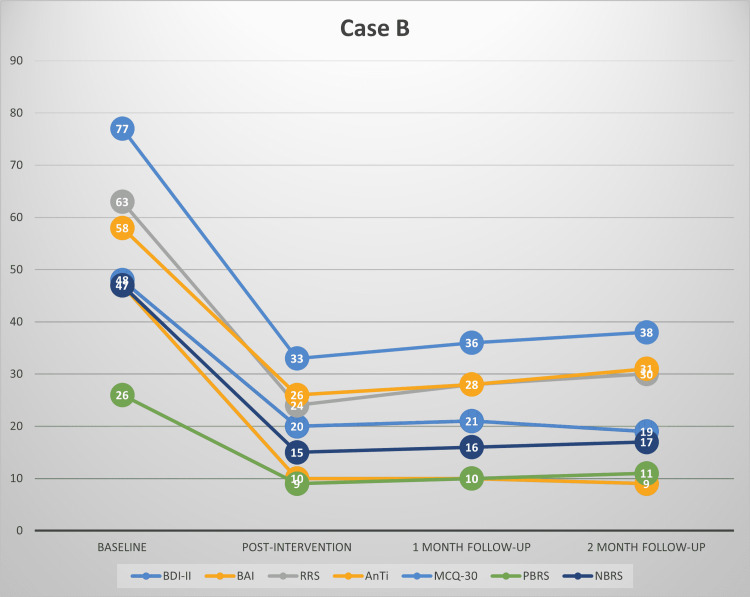
Scores of case B on BDI-II, BAI, RRS, AnTi, MCQ-30, PBRS, and NBRS across baseline, post-intervention, one-month follow-up and two-month follow-up BDI-II: Beck's depression inventory-II, BAI: Beck’s anxiety inventory, RRS: Ruminative Response scale, AnTi: anxious thoughts inventory, MCQ-30: metacognitions questionnaire-30, PBRS: Positive Beliefs about Rumination scale, NBRS: Negative Beliefs about Rumination scale

**Figure 3:**
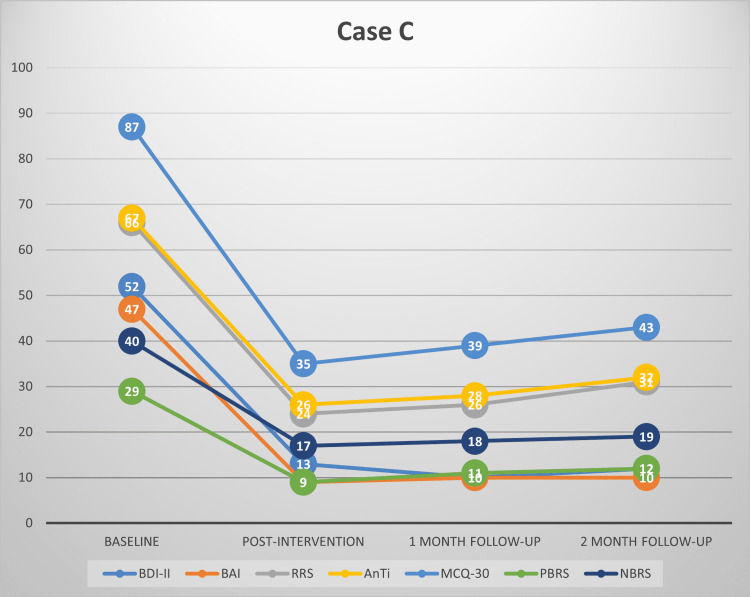
Scores of case C on BDI-II, BAI, RRS, AnTi, MCQ-30, PBRS, and NBRS across baseline, post-intervention, one-month follow-up and two-month follow-up BDI-II: Beck's depression inventory-II, BAI: Beck’s anxiety inventory, RRS: Ruminative Response scale, AnTi: anxious thoughts inventory, MCQ-30: metacognitions questionnaire-30, PBRS: Positive Beliefs about Rumination scale, NBRS: Negative Beliefs about Rumination scale

**Figure 4:**
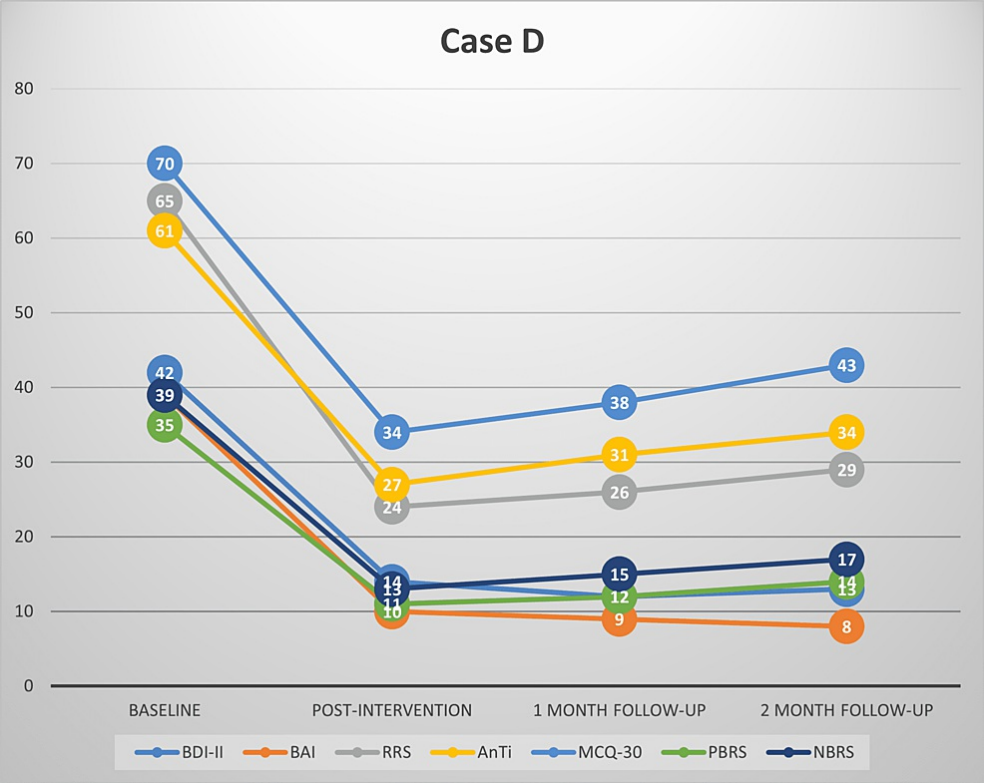
Scores of Case D on BDI-II, BAI, RRS, AnTi, MCQ-30, PBRS, and NBRS across baseline, post-intervention, one-month follow-up and two-month follow-up BDI-II: Beck's depression inventory-II, BAI: Beck’s anxiety inventory, RRS: Ruminative Response scale, AnTi: anxious thoughts inventory, MCQ-30: metacognitions questionnaire-30, PBRS: Positive Beliefs about Rumination scale, NBRS: Negative Beliefs about Rumination scale

This study’s findings provide evidence for the effectiveness of MCT among patients with depression and comorbid anxiety symptoms in the Indian context. This is complementary to our recent review highlighting the preliminary evidence for the effectiveness of MCT in various psychiatric disorders [32]. Substantial reductions were observed in depressive and anxiety symptoms in our patients at post-treatment and follow-up. MCT also brought a significant improvement in worry and rumination, along with a reduction in the underlying dysfunctional metacognitive beliefs. This redemonstrates that by targeting dysfunctional metacognitive beliefs about worry and rumination, one can treat symptoms of depression and anxiety. Each participant’s scores on all the primary outcome measures have been plotted in Figures 1-4, respectively.

At post-intervention, all the participants had large reductions in symptoms of depression and anxiety, along with marked improvement in the severity of rumination and worry, and a significant reduction in their dysfunctional metacognitive beliefs about rumination and worry. At post-intervention, two participants had mild depression, one had moderate depression, and one had minimal depression based on their BDI-II scores. All the participants achieved ≥50% reduction in the severity of depressive symptoms compared to baseline, thus meeting the criteria for clinically significant change. The reduction in depression severity was between 56% and 75% for all the participants at post-intervention. Using the Seggar criteria for significant change [25], three participants were classified as 'recovered', while one met the criteria for clinically significant improvement.

Similar findings were also obtained for anxiety symptoms. All the participants had large reductions in anxiety severity compared to baseline and had only mild anxiety symptoms post-intervention. All of them had a >50% reduction in anxiety symptoms as assessed using BAI. Anxiety symptom reduction for the participants ranged between 74% and 80% post-intervention. Applying the Westbrook criteria [25], all the participants met the criteria for ‘recovery’ post-intervention.

Clinically significant changes were also observed in the participants’ rumination and worry severity, as well as dysfunctional positive and negative metacognitive beliefs about worry and rumination. Three participants achieved a >50% reduction in the severity of their rumination, worry, and positive and negative metacognitive beliefs about rumination and worry.

At follow-up assessments, all the participants continued to meet the criteria of ≥50% reduction in depressive and anxiety symptoms. Treatment gains persisted during the follow-up period. Using the criteria of Seggar et al. [24], the three participants who had achieved recovery status post-intervention on BDI-II remained recovered, while one participant who had met the criteria for clinically significant improvement at post-intervention remained improved. When the criteria of Westbrook and Kirk [25] were applied, all the participants could be classified as ‘recovered’ on BAI at one month and two-month follow-ups.

Similarly, reductions in worry and rumination, and positive and negative metacognitive beliefs about worry and rumination remained clinically significant (≥50% reduction) for three of the participants at one-month and two-month follow-ups.

Our findings are consistent with previous work examining the benefits of MCT in managing depressive and anxiety symptoms [27,33]. Our study participants were able to acquire new skills that helped them identify perseverative thought cycles following low mood, were able to abandon these tendencies, and found this to be helpful in reducing their depressive and anxiety symptoms.

There are some notable limitations to our work. A case series design with a very small sample size precludes generalizing our findings to different clinical and socio-cultural contexts. We used strict selection criteria, which limits the ecological validity of the study. We relied on self-reported questionnaires, which may have biased the measurements of study variables. The primary author delivered the therapy and rated the patients on all the study outcomes, which can inadvertently bias the results further. The effect of therapist factors on the outcome could not be ruled out. We did not assess the intervention's fidelity and the patients' adherence to the homework assignments. The follow-up period was quite short, making it difficult to estimate the intermediate and long-term success of MCT.

## Conclusions

Overall, this exploratory study provides initial affirmation in support of MCT in the Indian context and warrants its further evaluation in managing symptoms of depression, anxiety, worry, rumination, and dysfunctional metacognitive beliefs about rumination and worry. Additionally, our study also yields some insights into the nature of metacognitive beliefs held by patients experiencing depression and anxiety, which may be unique to the local culture. MCT appeared to be associated with substantial improvements in symptoms of depression and anxiety simultaneously over a relatively short treatment duration. This is important in the Indian setting, given the scarcity of trained psychotherapists, poor access to treatment, and skewed therapist-patient ratio. MCT is a promising, cost-effective, and time-efficient approach compared to other psychotherapeutic modalities like CBT. However, large studies with robust methodological designs are needed to systematically test the effectiveness of MCT before it can be widely adopted in clinical practice.
